# The Future Prospects of Immune Therapy in Gastric and Esophageal Adenocarcinoma

**DOI:** 10.3390/jcm5110100

**Published:** 2016-11-14

**Authors:** Walid L. Shaib, Jean Paul A. Nammour, Harpaul Gill, Mayur Mody, Nabil F. Saba

**Affiliations:** 1 Department of Hematology and Oncology, Winship Cancer Institute, Emory University, 1365 Clifton Rd NE, Atlanta, GA 30322, USA; nfsaba@emory.edu; 2 Department of Medicine, Balamand University, Beirut 1300, Lebanon; jeanpaul.nammour@gmail.com; 3 Department of Internal Medicine, Emory University, Atlanta, GA 30322, USA; harpaul.s.gill@emory.edu (H.G.); mdmody@emory.edu (M.M.)

**Keywords:** esophageal cancer, immune therapy, progress

## Abstract

The prognosis of esophageal cancers is poor and novel approaches are urgently needed. Despite improvements in outcomes with transtuzumab and ramucirumab, these improvements added an average of only 2 to 3 months with a median overall survival reported to be around 1 year. Comprehensive genomic sequencing has defined some molecular alterations with potential targets, but the majority of patients still do not benefit from druggable targets. Breakthroughs in immune checkpoint blockade have provided new therapeutic options in many cancers. Programmed death ligand 1 (PDL1) overexpression, a possible biomarker predicting response to immune checkpoint inhibitors, approaches forty percent in esophageal and gastric cancers. Translational and molecular studies have shown that esophageal cancers are possible candidate malignancies for immune checkpoint inhibition. In this review, we plan to highlight the mechanisms, preclinical, and early clinical data that provide insight on the role of immune therapeutics in esophageal cancers.

## 1. Introduction

Esophageal cancer prevalence has been on the rise with a decline in incidence of gastric cancers [1,2]. The estimated new cases for esophageal and gastric cancers are 16,910 and 26,370, respectively, [2] projecting an urgent need for novel treatment approaches, as the majority of patients present with advanced disease. Options for treatments in the first line settings are limited to the platinum/5-fluoropyrimidine (5FU) backbone, which results in modest survival benefits in patients with good performance status [3]. This benefit was further enhanced by 2.8 months survival difference when adding transtuzumab to the chemotherapy backbone in the specific population of Her2/neu overexpression [4], which accounts for 20%–30% of esophagogastric adenocarcinomas [5,6]. In the second line treatment setting, vascular endothelial growth factor receptor (VEGF) inhibition with ramucirumab (a fully human monoclonal antibody (IgG1) targeting VEGFR2) as a monotherapy [7], or in combination with paclitaxel [8], resulted in a median overall survival (OS) of 9.6 months. This target was also studied in the first line setting with bevacizumab with a reported median progression-free survival (PFS) (6.7 vs. 5.3 months; *p* = 0.0037) and overall response rate (ORR) (46.0% vs. 37.4%; *p* = 0.0315), with significant improvement for the bevacizumab arm versus placebo. The primary objective, which was the median OS of 12.1 months with bevacizumab plus fluoropyrimidine-cisplatin vs. 10.1 months with placebo plus fluoropyrimidine-cisplatin, did not reach significance. The same cytotoxic chemotherapy backbone combined with ramucirumab is currently under investigation in the first line setting (RAINFALL). These survival numbers remain poor, urging the development of more effective treatment approaches that are needed for esophageal and gastric adenocarcinomas.

Immune-editing describes the changes in the immunogenicity of the cancer due to the anti-tumor response of the immune system, resulting in the emergence of immune-resistant variants. Immune-editing has three steps: elimination, equilibrium, and escape. The growth of neoplastic cells causes changes in the microenvironment and local damage. Immune cells, such as natural killer (NK) cells and T cells secrete INF-gamma, cytokines (CXCL9-10-11), and angiogenesis inhibitor factors in an attempt to inhibit tumor proliferation, in addition to chemokine secretions to enhance further immune cell recruitment. Tumor cell debris is processed by dendritic cells (DC), drained to neighboring lymph nodes, and presented to T cells. These then generate antigen-specific CD4+ and CD8+ T cells. During this initial elimination phase the immune system is able to recognize tumor specific antigens. Subsequently, a state of equilibrium consisting of a balance between the CD8+ T cells, DCs, as well as tumor cells exists. When tumor cells become active and proliferate, they “escape” the immune system. Immune therapy and check point inhibitors (CPI) are novel agents known to reverse this escape mechanism; CPI have been approved in melanoma, lung, and renal cell carcinomas and are considered breakthrough treatments in the clinical management of these cancers. CPI are being investigated in a number of malignancies, including squamous cell carcinoma of the head and neck and esophageal cancer, with a plan to isolate specific markers that might predict response and survival benefits to specific cancer populations such as the microsatellite instable colon and gastrointestinal malignancies [9]. The approval of these drugs has shifted the paradigm in these cancers and has raised interest in studying these compounds in different malignancies. The major approaches to modulate the cancer immunity came from the inhibitory antibodies to immune checkpoints, such as cytotoxic T-lymphocyte antigen 4 (CTLA4), programmed cell death protein 1 (PD1), and programmed death ligand 1 (PDL1). Cancer vaccines and adoptive T cell immunity are also areas of focus after several early phase trials have shown some promising activities in esophageal and gastric cancers. 

There is increased interest in developing immunotherapeutic strategies for the treatment of esophagus and gastric cancers. There are multiple targets in the process of immune-editing and immune activation that will be reviewed. In this paper, we are focusing on checkpoint pathways involving PD1, PDL-1, and CTLA4 in the treatment of esophageal and gastroesophageal cancers.

## 2. Pathways of Immune Targets

### 2.1. Adoptive Cell Immunity

Tumor-specific T cells are collected from patient’s blood and amplified in vitro. These cells are subsequently re-infused into the patient in large numbers. Different types of cells can be isolated, including activated killer cells, and several others that have been tested in gastric cancer such as tumor-infiltrating lymphocytes (TILs) [10]. Kono et al. randomized 44 patients with advanced gastric cancer with tumor-associated lymphocytes (TAL) with or without chemotherapy [11]. Patients with increased TAL had a survival advantage over those with no TAL cancers. Accumulating evidence shows a correlation between tumor-infiltrating lymphocytes (TILs) in cancer tissue and a favorable prognosis in various malignancies. 

### 2.2. Antigen Presentation and Vaccine Peptides

Vaccines, through activation of T cells, potentiate the immune response to target cancer. Antigen presenting cells (APC) would present tumor antigens to the cytotoxic T cells. These cells bind to specific MHC bound tumor cells and initiate an “immune” reaction against the tumor cells. One gene that was tested in gastric cancer is the *MAGE* (melanoma antigen-encoding gene) gene, which was first identified in melanoma, and has been found to be expressed in different solid tumors. It is also expressed in normal cells, without a known role. MAGE transcribes protein antigens targeted by CD8+ T cells, and in turn, the cytotoxic activity of the T cells is enhanced. Gastric cancers express MAGE (38%) [12]. Enhancing the expression of MAGE can further enhance immunity. MAGE expression can be induced by Helicobacter pylori [13]. In a preclinical setting where a nano-vaccine loaded with a MAGE-3 peptide was used, enhancement of the immune response in a mouse model of gastric cancer was observed resulting in tumor regression [14]. Other vaccine peptide developments could target tumor related antigens like *HER2/neu*, carcinoembryonic antigens, and even viral antigens such as HPV (Human papilloma virus), to enhance the immune response. One target that is actively being studied in a phase I clinical trial is the DKN-01, which is a humanized monoclonal antibody (Mab) with neutralizing activity against Dkk-1. This is currently being developed as an anti-neoplastic agent in squamous cell carcinoma and adenocarcinoma of the esophagus (NCT02013154) [15].

### 2.3. Checkpoint Inhibitors

**Figure 1:**
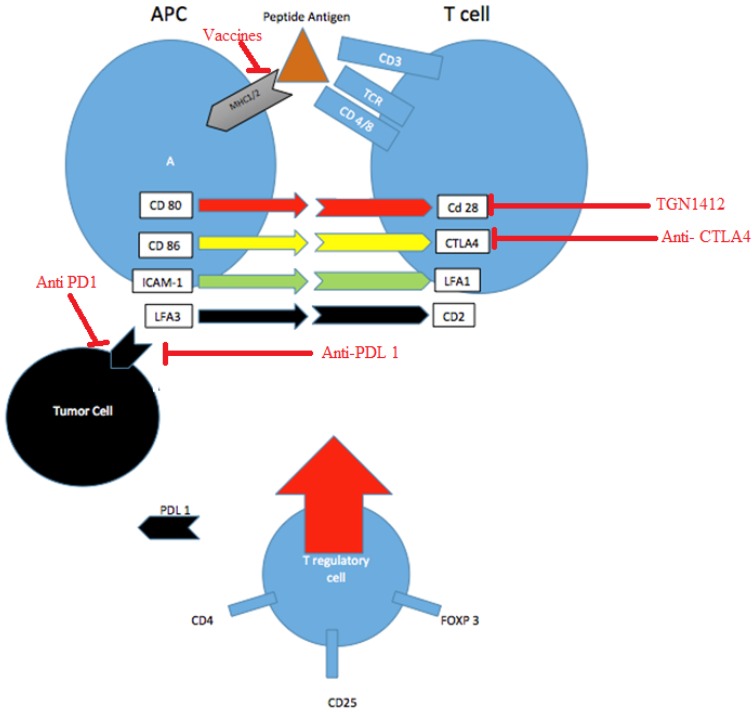
T cells recognize antigens presented to the T-cell receptor (TCR) as antigen peptides within the major histocompatibility complex of the Antigen Presenting Cells (APC) and tumor cells. Tumor cells usually stimulate a MHC I- CD8+ T cell response. Both the APC and tumor cell engage a co-stimulatory effect of the T cell through the CTLA4 and CD28 binding to the APC ligands (CD80 also called B7-1, and CD86 also called B7-2). Another dependent stimulatory effect is the intercellular adhesion molecule-1 (ICAM1) and leukocyte function-associated antigen (LAF3) on the APC that bind to the LFA-1 and CD2 respectively. The formation of PD1/PDL 1 or B7-1 receptor /PDL 1 ligand complexes transmits an inhibitory signal which reduces the proliferation of the CD8+ T cells. Engagement of PDL 1 with its receptor PD1 on T cells delivers a signal that inhibits TCR-mediated activation of IL-2 production and T cell proliferation. T-regulatory cells (Tregs) serve as checkpoints and suppress the expansion of T cells directed against self-antigens. A: Antigen Presenting Cell (APC) TGN1412 was developed as a CD28 superagonist, but failed to reach clinical trials due to multiorgan failure. Vaccines are being developed to target specific MLH (MutL Homolog) sequences.

The activity of the immune system is regulated in such a way that avoids excessive over-activation to prevent tissue damage to healthy cells. Several checkpoints are involved in this balanced process. CTLA4 and programmed cell death protein-1 (PD1) are inhibitory receptors expressed by the T cells. Targeting these receptors on the T cells by antibodies blocks their inhibitory potential and further activate cytotoxic T cells. This leads to further activation of these cells against the tumor cells [16,17,18]. Figure 1 illustrates the various interactions of immune checkpoints.

**A- CTLA4:** It is a T-cell receptor (CD152) that belongs to the immunoglobin superfamily. CTLA4 shares similarities with the T-cell co-stimulatory protein CD28, and gets activated when binding to CD80 (B7-1) or CD86 (B7-2) on antigen-presenting cells [19]. CD4+ helper T cells activated by CTLA4 inhibit T cell activity, whereas in CD4+ T regulatory cells (Treg), increases T cell activity. Thus, the net effect is immune tolerance [20]. This is actively being studied in a phase II clinical trial that involves gastric and gastroesophageal junction (GEJ) malignancies by utilizing ipilimumab as the CTLA4 inhibitor (NCT01585987) [21]. In a phase II trial, Tremelimumab, a fully humanized anti-CTLA4 monoclonal antibody, was tested in the second-line setting with an enrollment of 18 patients with gastric cancer. The objective response rate was 5%, and the median overall survival (OS) was 4.8 months. This is comparable to taxanes in second line treatment [22]. Although this trial reported suboptimal results, it resulted in a growing interest in combining two checkpoint inhibitors after the approval of the CTLA4 and PD1 inhibition in melanoma [23]. Preclinical data proved an increased immune enhancement for the dual blockade of PD1 and CTLA4 when compared with single receptor blockade [24,25].

**B- PD1/PDL-1:** PD1 is an inhibitory receptor belonging to the CD28-B7 family. It binds to two ligands, PDL1 and PD-L2, resulting in down regulation of the T cell immune response. Therefore tumors that are capable of mediating a strong PDL1 expression will cause immune suppression and result in poor prognosis. PD1 is expressed in T, B, and NK cells. The higher the expression of PD1 and PDL1 in the cancer, the worse the outcome. In addition, the expression of PD1 was significantly correlated with poor clinically pathological parameters such as the depth of invasion, lymph node metastasis, and distant metastasis [26,27]. The Cancer Genome Atlas Research Network analyzed the molecular characteristics of gastric adenocarcinoma and identified four subtypes: Epstein–Barr virus (EBV-15%), MSI (microsatellite instable), MSS (microsatellite stable), and tumors with chromosomal instability. The EBV subgroup had elevated PDL1 expression, suggesting a better immune reaction against gastric cancers when immune checkpoint inhibitors are used.

The phase I/II, open-label CheckMate-032 study evaluated nivolumab and nivolumab/ipilimumab combination in patients with solid tumors. The experience in patients with gastroesophageal junction and gastric adenocarcinoma was reported in the cohort receiving nivolumab monotherapy. Fifty-nine patients were enrolled in the third line treatment setting. A median of 4 doses was received and a response was reported in 12% (7/58; 1 complete response, 6 partial responses); 12 patients (21%) had stable disease. The reported 1-year survival was 38% (95% CI, 23.2–52.7). PDL1 expression was reported for 39% of the tumor samples (<1% cutoff), and the response rate for PDL1-positive and -negative tumors were 18% and 12%, respectively. All side effects were expected and no treatment related deaths were observed (NCT01928394) [28]. In the phase III study, Checkmate 141, OS was significantly improved in patients who received single agent nivolumab when compared to single-agent investigator’s choice of chemotherapy (12-month OS of 36% nivolumab vs. 17% chemotherapy) in platinum refractory recurrent or metastatic head and neck squamous cell carcinoma (HNSCC). This study enrolled 240 patients. Participants were either given nivolumab (3 mg/kg IV every 2 weeks) or various other second line chemotherapy options, which included methotrexate, docetaxel, or cetuximab. Median OS was 7.5 months for nivolumab compared to 5.1 months for chemotherapy (HR 0.70; *p* = 0.010). This was the first randomized clinical trial to demonstrate improved OS for patients with platinum refractory HNSCC [29].

Pembrolizumab is another monoclonal antibody designed to block the interaction between PD1 and its ligands PDL1 and PD-L2. Muro et al. investigated the safety and activity of pembrolizumab in gastric cancer patients in a phase Ib trial [30]. Patients with advanced gastric or GEJ cancers were screened (*N* = 165) and 40% (65 patients) were PDL1 positive (defined as PDL1 staining in stroma or ≥1% of tumor cells), 39 of whom were treated with Pembrolizumab (10 mg/kg) every 2 weeks. A decrease in tumor burden was reported in 41% of patients. The ORR was 32% in Asian patients and 30% in non-Asian patients. The treatment was very well tolerated [30]. Furthermore, preliminary evidence of a relationship between PFS, ORR, and PDL1 expression was observed. Another phase Ib study of pembrolizumab in subjects with gastric cancer (KN012) was designed to investigate if pembrolizumab monotherapy in gastric and GEJ adenocarcinoma patients at any line of treatment improves outcomes. Thirty-nine patients enrolled (19 from clinical trial sites in Asia and 20 from trial sites outside Asia) and selected positive for PDL1 by immunohistochemistry (defined as staining in ≥1% of tumor cells or any stroma cells using a prototype assay). Primary endpoint was RR (Response rate) and it was reported at 30.8% (range: 17.0% to 47.6%; all partial responses). The response was similar in patients from Asia and outside of Asia. The gastric cancer proof-of-concept from Keynote 012 data was obtained in subjects with a PDL1 positive expression only; no data is currently available regarding the performance of pembrolizumab in subjects without a detectable PDL1 expression. Due to the limited treatment options currently available, second line subjects without a detectable PDL1 expression may still benefit from pembrolizumab. In the multi-cohort, phase Ib KEYNOTE-028 (NCT02054806) trial, patients had advanced squamous cell carcinoma or adenocarcinoma of the esophagus or GEJ, and PDL1 expression in tumor or stroma was determined centrally by IHC. The drug was proven to be safe with the known autoimmune side effects and a median duration of response of 40 weeks ranging from 24.1 to 46.1 weeks. Analysis of the relationship between PDL1 expression level and antitumor activity is ongoing. This is the smallest of the studies reported thus far, and had the highest response rate in 7 of the 23 patients enrolled, who were treated for metastatic squamous cell carcinoma and adenocarcinoma of the esophagus. Of the 23 patients enrolled, 12 had some response and the median duration of response had not been reached (5.5–11.8 months). These patients were enrolled in a larger cohort of a phase I trial that included other patients with advanced solid tumors [31,32]. Another trial, KEYNOTE 180 (NCT02559687), is a phase II study currently accruing patients, and will assess the effects of pembrolizumab on ORR in patients with advanced/metastatic adenocarcinoma or squamous cell carcinoma of the esophagus or advanced/metastatic Siewert type I adenocarcinoma of the GEJ previously treated with two standard therapies [33].

Another human IgG1 monoclonal antibody against PDL1, MEDI4736, showed activity in a phase I clinical trial against multiple solid tumors. This is actively being tested in phase Ib/II trials combining MEDI4736 alone or in combination with Tremelimumab (NCT02340975) [30]. Avelumab, a new PDL-1 inhibitor, demonstrated clinical activity as a second-line and maintenance therapy for patients with unresectable gastric or GEJ cancer. In the phase Ib study, 55 patients were enrolled (20 as chemotherapy naïve, 35 who had stable disease on chemotherapy). Of the patients who received avelumab as maintenance after first-line chemotherapy, one had a complete response and three had partial responses, with an ORR (Overall Response Rate) reported at 7.3%. In the second-line setting, the ORR reached 15%. Stable disease rates were 35% in the second-line setting and 47.3% in the maintenance cohort for avelumab treated patients. Avelumab was administered at 10 mg/kg every 2 weeks; the adverse events were similar and comparable to pembrolizumab and nivolumab [34]. Table 1 summarizes the completed and ongoing trials for esophageal, HNSCC and gastric cancers. 

## 3. Conclusions

Cancer immunotherapy has now come to age with the recent approval of immune checkpoint therapies in advanced melanoma, renal cell carcinoma, and non-small cell lung cancers. In esophageal, gastric, and gastroesophageal cancers, several promising steps have been made, but more is still needed. The prevalence of esophageal cancer, in particular, is on the rise, and patients with advanced disease have an overall poor prognosis. Options for therapy are limited in this patient population. In this review, we have highlighted several trials that have demonstrated the activity of immunotherapy, and in particular CPI, on various GI malignancies. Muro et al. investigated the safety and activity of pembrolizumab in gastric cancer patients in a phase Ib trial with promising results. The Checkmate 141 trial is the first phase III randomized study to show improved OS with nivolumab in patients with advanced HNSCC. These studies exemplify the clear activity that CPI have in gastric as well as head and neck malignancies. Thus patients with advanced esophageal carcinoma would likely also stand to benefit from treatment with these therapies, especially when subgroups of esophageal cancers of patients enrolled in all advanced solid tumors phase I trials, are showing promising benefits. The phase II trial has demonstrated that Nivolumab has meaningful activity and a manageable safety profile in patients with esophageal cancer and a phase III study is commencing. Ongoing trials such as KEYNOTE 180 (NCT02559687) will also be investigating the effects of immunotherapy (pembrolizumab) on advanced esophageal carcinoma.

With better understanding of these cancers’ biology, a number of studies have established higher expression rates of immune checkpoints in cancer tumor tissues versus normal control tissues. We know more about the checkpoint inhibitors with regards to their mechanism of action and their side effects. These therapies help in priming the microenvironment for the development and progression of cancer. Further exploration and assessment of the biomarkers, such as the expression of the PD1 or PDL1, are needed to select the group that benefit most from these inhibitors. Dual check point inhibition, combined chemotherapy with checkpoint inhibitors, and combined checkpoint inhibition with other investigational immunotherapeutic options are needed to further enhance the immune system and prime it against cancer. Molecularly selected patients will not only help individual patients in a personalized approach, but will also help decrease side effects to patients that might not benefit from the treatment, as well as help decrease the cost of care. Future clinical trials should, therefore, include a substantial translational research component, investigating potential biomarkers and mutational aberrations for better targeting of cancer.

## Figures and Tables

**Table 1:** Completed and ongoing trials (Phase I–III) investigating immune therapy in esophageal, HNSCC, and gastric cancer.

Trial	N	Phase	Regimen	Cancer Subtype	OS (Months)	1 Year OS (%)	ORR (%)
Ralph et al. [22]	18	II	Tremelimumab	Gastric and Esophageal Adenocarcinoma	4.8	-	5
Le et al. [28]	59	I	Nivolumab	Gastric and Gastroesophageal adenocarcinoma	-	36	14
Muro et al. [30]	39	Ib	Pembrolizumab	Gastric adenocarcinoma	-	-	32 in Asian population. 30 in non-Asian population
NCT02559687 (ongoing) [33]	100 (estimated)	II	Pembrolizumab	Esophageal Adenocarcinoma and Squamous Cell Carcinoma	-	-	-
NCT01585987 (ongoing) [21]	114	II	Ipilimumab	Gastric and Gastroesophageal adenocarcinoma	-	-	-
